# Clinical Experience Using a Dual-Layer Amniotic Membrane Allograft on a Posterior Upper-Thigh Pressure Ulcer

**DOI:** 10.3390/reports8040199

**Published:** 2025-10-06

**Authors:** Kirk Mitchell

**Affiliations:** Compassionate Concierge Physicians, Longmont, CO 80501, USA; doctormitchell54@gmail.com

**Keywords:** dual-layer allograft, amniotic membrane tissue allograft, chronic wound, pressure ulcer, chronic ulcer, pressure injury

## Abstract

**Background and Clinical Significance**: The objective of this case study is to report on the clinical outcomes of a hard-to-heal posterior upper-thigh pressure ulcer when managed with a sterile human amniotic membrane tissue allograft. **Case Presentation**: Retrospective case data of a patient who received five applications of barrera^TM^ between February 2024 and April 2024 as part of their care regimen for a chronic pressure ulcer was obtained from a single wound care group. Data evaluated consisted of past patient medical history, concomitant medications, previous wound care treatments, wound type, wound size, adjunctive wound therapies and wound outcomes post allograft. The chronic pressure ulcer, measuring at 10.5 cm^2^ prior to allograft application, achieved complete closure at the last observation post the final, fifth application. Wound size attenuation was seen as early as 1 week post initial allograft application. No adverse events or complications related to barrera^TM^ were observed. **Conclusions**: Results suggest that the application of dual-layer allografts in the context of chronic ulcers may represent a safe and effective wound management utility.

## 1. Introduction and Clinical Significance

Acute pressure injuries can rapidly become chronic over time, particularly within patient populations suffering from age-related health issues, mobility ailments, diagnosed with chronic conditions, surgical patients, and those who reside within hospitals, long-term care facilities, nursing homes, etc. [1]. Many individuals in such situations encounter both economic and logistical barriers that block access to appropriate care, thereby complicating regular wound management and decreasing regimen compliance, ultimately leading to diminished patient outcomes. As susceptibility arises, the need for appropriate wound management techniques arises.

Emerging advanced wound care technologies have focused on cellular, acellular and matrix-like products (CAMPs) to support wound management. Among these CAMPs are placental allografts, which have arisen as a promising option due to the amniotic membrane’s rich, native composition of extracellular matrix (ECM) components [2]. Currently, placental-derived allografts are widely used in wound care settings and are commonly processed as single- or multi-layer constructs [3]. For the patient presentation within this case study, an established dual-amnion graft technology, barrera^TM^, was applied after conservative treatments failed.

## 2. Case Presentation

The retrospective data presented is from a single wound care center (Compassionate Concierge Physicians, CO, USA), dating from February 2024 to April 2024. This report is centered on a 65-year-old, white, non-Hispanic, non-Latino male with no known drug allergens. He is a non-smoker, has a medical history of multiple sclerosis with significant immobility, potential for infection and is wheelchair bound. Progress reports note that the patient had a wound history of bilateral chronic upper-thigh and buttocks decubitus ulcers that have been undergoing wound care for many months. His wounds had been previously managed using a preferred wound care cleanser, non-adherent, petroleum-based gauze dressings, and absorbent foam dressings.

As a result of continued immobility, the patient developed a category 2 pressure ulcer on his left posterior upper thigh, initially separated into three oval-shaped wounds. At 1 week prior to allograft application for this wound, the measurement was presented as 4.2 cm × 2 cm (length × width), with mild, occasional pain and mild tenderness, especially when seated in his wheelchair. Although the wound is divided into three parts, measurements were recorded collectively as there was minimal spacing between the three parts. Additionally, the collective measurement encapsulates the total wound burden area.

Imaging with a point-of-care fluorescence wound imaging device was determined medically necessary to evaluate for bacterial bioburden that could inhibit wound size regression and to target debridement within high-bacterial-burden areas. The MolecuLight device was placed at an appropriate distance (approximately 10 cm) from the left posterior upper-thigh wound with the required dark conditions to ensure proper ambient lighting. A standard image and a fluorescent image were taken of the wound. Results depicted that no significant bacterial burden was evident, thus confirming the wound care plan.

Immediately prior to the initial barrera^TM^ (RegenTX Partners, LLC, an affiliate company of Tiger BioSciences, San Antonio, TX, USA) allograft application, the ulcer measured 3.5 cm × 3 cm (length × width), was still separated into three oval-shaped wounds, had positive mild tenderness, no discharge and minimal erythema. The wound was debrided for 15 min by soaking with a wound care cleansing solution; additionally, surrounding tissue and wounds were scrubbed and cleansed using gauze soaked with the wound care solution for >60 s to remove devitalized tissue. After this, barrera^TM^ amniotic membrane allografts were applied to the wound bed of the posterior thigh with the assistance of saline to cover the entire wound bed. The wound was then dressed with a non-adherent, petroleum-based gauze dressing and an absorbent foam dressing which were secured with a thin border of clear tattoo tape. It was noted that the patient tolerated the procedure well, and there were no complications reported throughout the procedure.

**Figure 1:**
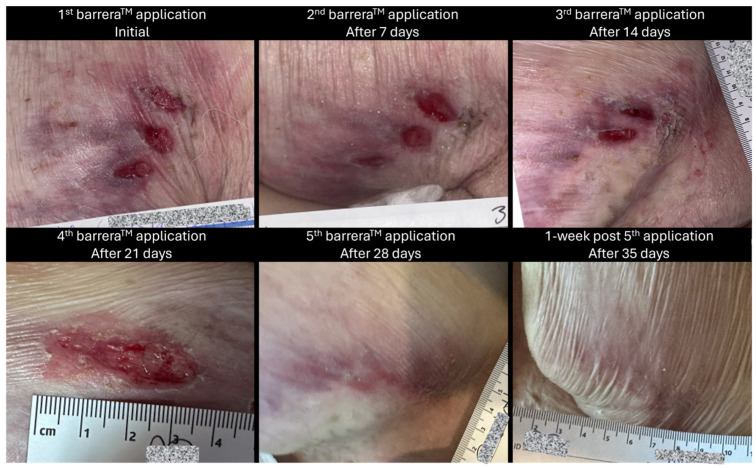
The presented is a pictorial representation of the left posterior upper-thigh wound at weekly intervals. This image depicts the change in wound dimensions from the 1st allograft application to 1 week post final, 5th application. Subject identifiers have been redacted within the images for privacy purposes.

**Figure 2:**
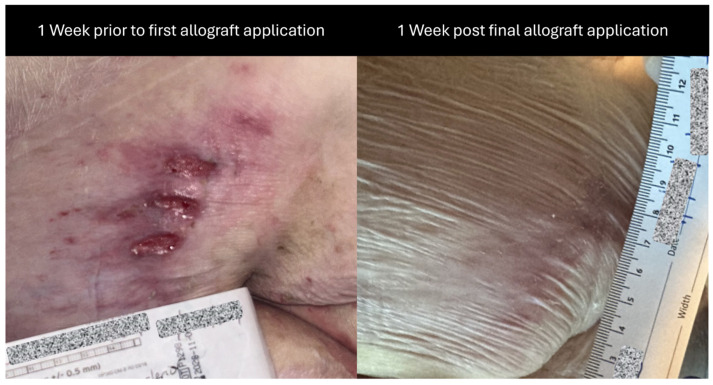
The figure presents a pictorial representation of the left posterior upper-thigh wound at pre-allograft and post-allograft application timepoints. This comparison image depicts wound changes seen from 1 week prior to allograft usage to 1 week post final, 5th application. Subject identifiers have been redacted within the images for privacy purposes.

Weekly wound care and barrera^TM^ allograft placements were provided by qualified providers. In addition, the patient’s daily aide was instructed to perform wound care and dress changes every other day. The patient received a total of five applications of barrera^TM^ between February 2024 and April 2024 as a part of their care regimen. A pictorial representation of the wound at different time intervals throughout the course of care can be seen in Figure 1 and Figure 2.

### Quantitative Measures

**Figure 3:**
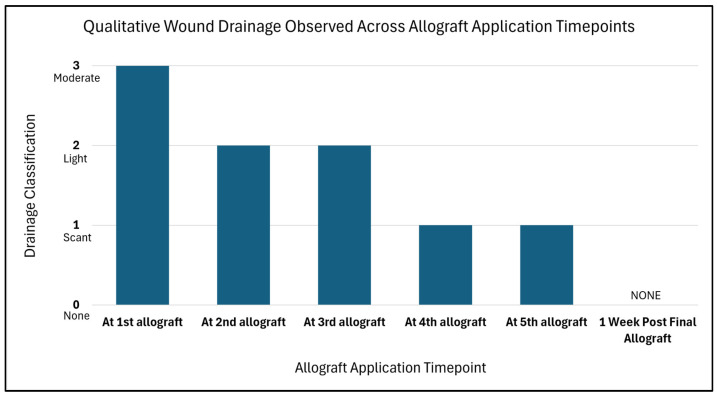
Within this figure qualitative wound drainage classifications are observed across allograft application timepoints. The figure depicts an exudate trend, which is indicative of a wound that is progressing towards a positive state over time. As the wound is progressing towards favorable outcomes, an overall reduction from moderate classification to none is noted. This can be further seen within the pictorial representations in Figure 1.

An average 2.1 cm^2^ change in the wound area per week, measured as the rate of healing, was seen from the first application of barrera^TM^ (immediately before allograft application, wound area: 10.5 cm^2^) to 1 week post final allograft application (wound area: 0 cm^2^). A corresponding change in the amount of drainage was also observed, going from moderate drainage to no drainage, suggesting a positive, healthy progression as seen in Figure 3.

**Figure 4:**
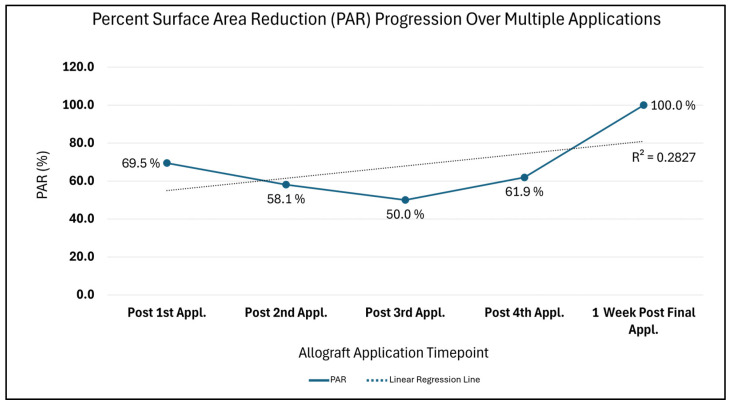
This figure presents Percent Surface Area Reduction (PAR) change as compared to the first allograft application timepoint. The trend in this figure depicts an overall improvement in percent surface area when compared to the initial SA size measurement at application timepoint 1, when the wound was measured at 10.5 cm^2^. PAR = [(Initial SA − Current SA) ÷ Initial SA] × 100.

**Figure 5:**
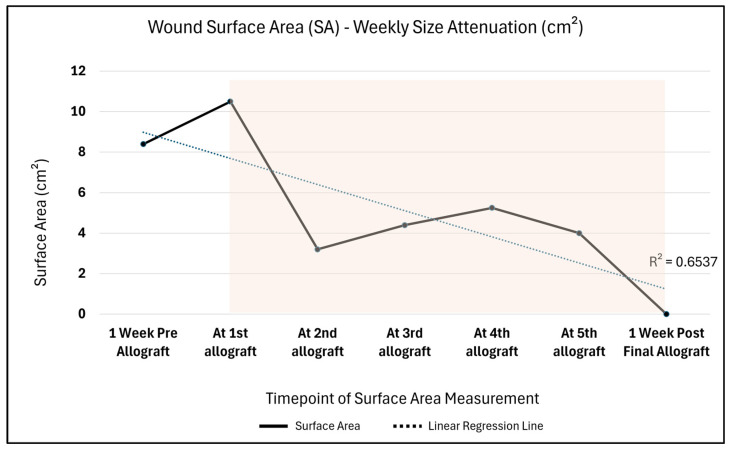
This figure illustrates the observed Wound Surface Area (SA) weekly size attenuation trend; with the shaded area highlighting the timepoints related to allograft application. The observed impact of a dual-layer allograft on a posterior upper-thigh pressure ulcer is depicted within the SA attenuation trend seen in this figure. It can be noted that the wound size had increased prior to the first placement of barrera^TM^. Post application of barrera^TM^, an overall measurement reduction was seen until the wound reached 0 cm^2^.

Additionally, the Percent Surface Area Reduction (PAR = [(Initial surface area − Current surface area) ÷ Initial surface area] × 100), with the initial wound area comparison timepoint being 10.5 cm^2^, depicts trends synonymous with a wound advancing towards 0 cm^2^ closure over time (Figure 4). A drastic 69.5% PAR immediately after the first allograft application is noted, after which a continued percent improvement trend is seen until 100% size reduction is reached. Likewise, in Figure 5, the comparison of the initial surface area (SA = length × width) to weekly SA measures also depicts a size attenuation trend. The slight upward trend seen at the third and fourth allograft application timepoints may be associated with the three oval-shaped wounds combining into one. The consolidation of the adjacent wounds may be attributed to friction or sheer associated with movement; however, the timepoint of this occurrence is unknown. Of note, there were no signs of adverse events nor infection during the course of care.

## 3. Discussion

Yearly, more than 2.5 million people in the United States develop pressure ulcers, which not only increases physical distress, discomfort and risks for infection, but also adds to an overall increase in health care utilization [4]. Recent hospital metrics and the literature have shown pressure ulcers to be the third most costly disease after cancers and cardiovascular diseases, with mortality rates being two to six times higher compared to other maladies [4]. Given their increasing emergence, it is not surprising that approximately 60,000 deaths annually are due to this complication, and risk factors such as diminished physical activity, decreased consciousness, incontinence, malnutrition and/or advanced age further contribute to the prevalence of these ulcers [4]. Once developed, pressure ulcers are commonly associated with decreased mobility, and, in more critical cases, severe infection or limb amputation—all negative outcomes that providers and patients aim to mitigate early on. Amniotic placental allografts act as supportive wound coverings, which have been shown to provide encouraging results as adjuncts to standard of care (SOC) for wounds such as pressure ulcers. When applied to ulcers of varying etiologies, dual-layer amniotic placental allografts may provide supplemental support during the body’s natural healing process. In addition, based on the prevailing scientific literature, they may offer anti-inflammatory effects, anti-microbial effects, minimize the need for more invasive interventions and prevent unnecessary hospitalization [5,6].

The human amniotic membrane is a thin collagenous membrane derived from the submucosa of the placenta. It is composed of collagen and the extracellular stromal matrix, both of which are critical for providing structural integrity in allograft-based wound coverings [5,7]. barrera™ is one such terminally sterile, dual-layer amniotic placental allograft, which may be used on hard-to-heal, full-thickness wounds. Through the manner in which barrera™ is minimally processed, the native physical integrity of the amnion layers is maintained. Its dual-layer configuration contributes to greater durability relative to single-layer amniotic allografts. In addition, the layering is associated with a slower degradation rate, such that the allograft remains intact longer than a single amnion layer [8].

This case highlights the clinical utility of barrera^TM^ in the management of a recalcitrant stage 2 pressure injury in a patient with complex care needs. The 65-year-old patient presented with a medical history of multiple sclerosis with severe immobility. While multiple sclerosis is not directly related to the causation of the wounds, the immobility and diminished body sensation complications associated with the disease substantially increased susceptibility to the development of pressure ulcers for this patient. Based on clinical progress notes, the patient’s predisposition to the development of chronic wounds, plus potential presence of external factors such as sheer forces due to repositioning movements, significantly hindered advancement towards wound size regression. In order to avoid future hospitalization, decrease risks of exacerbated infections, and to circumvent more aggressive forms of wound mitigation techniques, placental amniotic allograft application was preferred by both the patient and the patient’s provider team. In this context, the favorable response observed with the application of barrera^TM^ suggests it may offer meaningful clinical benefits as a valuable wound covering, which supports wound regression targets for recalcitrant wounds commonly encountered in immobile, high-risk geriatric populations.

This interval-based, objective analysis of wounds, albeit presented within this paper for an N of 1, contributes to the evidence-based allograft application approach. The SA and PAR measurements are key, complementary wound evaluation metrics, which are used herein to provide empirical clinical insight for the application of barrera^TM^. Surface area calculations provide a static snapshot of wound dimensions at particular time intervals, thereby depicting the absolute change over time, which can be compared to initial measurements, whereas dynamic PAR size metrics capture the percentage of change seen for the wound at varying time intervals, depicting a measure of responsiveness. This case study reflects trends supported by the previously published literature, which demonstrate that a 20–30% PAR change in wound size within the first 3 to 4 weeks following amniotic allograft application is a viable benchmark for wound response and prediction of progress towards favorable outcomes [9,10]. Additionally, the observed reduction in wound SA over time and decrease in wound drainage for the patient presented herein are indicative of progressive amelioration post application of barrera^TM^ to the care regimen.

It is acknowledged that limitations of this study include its retrospective design and the small, single-patient sample size. Larger patient cohort studies with SOC control groups are warranted to further define and assess the results of utilizing a dual-layer placental allograft.

## 4. Conclusions

The intent of this paper was to report on the clinical experience of using a dual-layer amniotic placental tissue allograft, barrera^TM^, in the management of a hard-to-heal, stage 2 pressure ulcer. This clinical presentation depicts the potential wound-protective properties of dual-layer allografts when applied to complex, recalcitrant wounds within a high-risk patient population, such as the elderly and those with mobility restraints. The paper further expounds upon the potential of placental allografts to facilitate positive clinical endpoint responses, which may improve a patient’s quality of life, decrease infection risks and overall limit the need for hospitalization.

## Data Availability

The data that supports the findings of the manuscript may be available from the corresponding author upon reasonable request that takes into account patient privacy requirements.

## Abbreviations

The following abbreviations are used in this manuscript:

CAMPs	Cellular, Acellular and Matrix-like Products
ECM	Extracellular Matrix
PAR	Percent Area Reduction
SA	Surface Area
SOC	Standard of Care
